# News or social media? Socio-economic divide of mobile service consumption

**DOI:** 10.1098/rsif.2021.0350

**Published:** 2021-12-01

**Authors:** Iñaki Ucar, Marco Gramaglia, Marco Fiore, Zbigniew Smoreda, Esteban Moro

**Affiliations:** ^1^ UC3M-Santander Big Data Institute, Universidad Carlos III de Madrid, Getafe 28903, Spain; ^2^ Department of Telematic Engineering, Universidad Carlos III de Madrid, Leganés 28911, Spain; ^3^ IMDEA Networks Institute, Leganés 28918, Spain; ^4^ Sociology and Economics of Networks and Services Department, Orange Innovation, Châtillon 92320, France; ^5^ Department of Mathematics, Grupo Interdisciplinar de Sistemas Complejos, Universidad Carlos III de Madrid, Leganés 28911, Spain; ^6^ Connection Science, Institute for Data Science and Society, MIT, Cambridge, MA 02139, USA

**Keywords:** digital usage gap, inequality, mobile phone data, development, privacy preserving

## Abstract

Reliable and timely information on socio-economic status and divides is critical to social and economic research and policing. Novel data sources from mobile communication platforms have enabled new cost-effective approaches and models to investigate social disparity, but their lack of interpretability, accuracy or scale has limited their relevance to date. We investigate the divide in digital mobile service usage with a large dataset of 3.7 billion time-stamped and geo-referenced mobile traffic records in a major European country, and find profound geographical unevenness in mobile service usage—especially on news, e-mail, social media consumption and audio/video streaming. We relate such diversity with income, educational attainment and inequality, and reveal how low-income or low-education areas are more likely to engage in video streaming or social media and less in news consumption, information searching, e-mail or audio streaming. The digital usage gap is so large that we can accurately infer the socio-economic status of a small area or even its Gini coefficient only from aggregated data traffic. Our results make the case for an inexpensive, privacy-preserving, real-time and scalable way to understand the digital usage divide and, in turn, poverty, unemployment or economic growth in our societies through mobile phone data.

## 1. Introduction

Inequality is a central societal problem, especially within rapidly expanding urban areas. While it is a crucial driver for economic growth [1], the progressive clusterization of workers, industries, companies and services in cities has a tremendous cost in terms of segregation and discrimination. This cost is not only economic: in the same city, different areas can have a 10- to 15-year imbalance in life expectancy and highly divergent education levels, with little chances of social mobility [2]. The design and successful implementation of policies to alleviate these problems require fine-grained, frequently updated information about income, education or inequality across metropolitan areas. However, most data sources employed today, such as population censuses or surveys, suffer from sparsity in population coverage or infrequent updating, hence they do not allow the swift evolution that urban societies experience nowadays to be followed. Thus, the traditional ways of understanding cities tend to explain what happened 5 years earlier rather than *nowcasting* or even predicting urban transformations.

In recent years, digital data have been proposed as an alternative source for socio-economic status (SES) inference [3–5]. The escalating use of mobile devices [6–9], social media [10] or credit cards [11] and the growing availability of pervasive satellite imagery [12,13] have allowed researchers to build SES models with unprecedented temporal and spatial resolutions. For example, income levels in urban areas were correlated with the unequal presence of trucks [14] or utilization of construction materials [15] extrapolated from imagery data. Similarly, the diversity in human mobility or social interactions observed in data from mobile phones or social media was found to be correlated with higher income [8,10]. However, while very successful in predicting SES in developing countries [7,9,12], these approaches are only moderately accurate in developed countries [8,16], where variances in the penetration of mobile phones, in the use of credit cards and in social segregation itself are more nuanced.

When considering economic and social inequality in wealthier countries, we argue that specific mobile services’ consumption may be a more suitable proxy for SES than other digital data considered to date. Indeed, a diffuse preference for particular mobile applications is a more subtle indicator than the sheer adoption of mobile digital technologies, as it connects to finer-grained user traits such as personal interests, digital skills or accessibility to paying services [17,18].

Several previous studies provide some evidence that corroborates our postulation. For instance, it has been hypothesized that mobile service usage can reveal the digital divide between different socio-economic, gender or age groups [17]. There is qualitative confirmation that mobile digital usage might exacerbate socio-economic inequalities given the impact that social media and online information resources have on the social, political and economic aspects of our society [18,19]. It is also known that time on some social platforms, watching videos or playing videogames [20] or news media consumption patterns [21] depend on users' SES, and that students’ performance is related to different patterns in their Internet usage [22,23]. All these studies suggest a significant disparity in how mobile services are consumed, even in developed economies where the technology access gap is not significant. Nevertheless, the limited scale and small granularity of existing studies do not allow a conclusive opinion to be formed on the magnitude of such a mobile application usage gap nor do they allow its repercussions on SES features to be understood.

In this paper, we present the first large-scale, quantitative study of the relationship between mobile service adoption and socio-economic inequality. To that end, we analyse nationwide data traffic measurements collected by the leading mobile operator in a major European country (France), and find fundamental imbalances in the relative usage of specific mobile applications by different income or education groups during particular time periods. More precisely, we focus on a time frame where individuals are most likely to be in their residential areas, which favours the matching of mobile phone usage with demographic data. In such intervals, the mobile service consumption gap is so profound that we can build fairly accurate models based on mobile traffic to estimate income, education level and economic inequality at high spatial resolution.

## 2. Results

**Figure 1.**
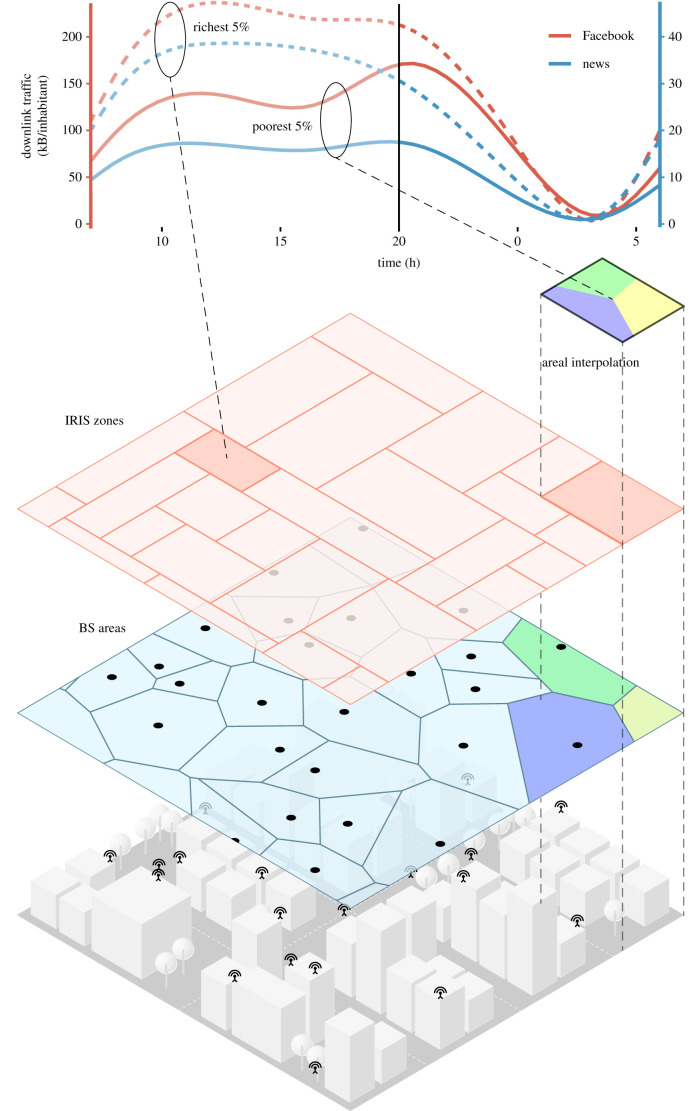
Areal interpolation infographic. The mobile traffic dataset comprises mobile service usage statistics for 25 000 geo-located base stations (BS; bottom layer). The coverage areas of BS are approximated by Voronoi polygons where mobile traffic is assumed to be uniformly distributed (middle layer). The mobile traffic is weighted and interpolated into French administrative areas (IRIS zones; top layer). The top plot depicts the average daily time series of downlink traffic per inhabitant at the richest 5% (dashed lines) and the poorest 5% IRIS zones in Paris for two representative mobile services: Facebook (red) and news (blue). As can be seen, time series of raw byte counts in the same area are highly correlated and reveal little information. However, the relative traffic generated by the two services in different areas exposes unique patterns that can be exploited for SES prediction.

Our data consist of around 3.7 billion time-stamped and geo-referenced records of the mobile traffic generated by different applications, such as YouTube, Facebook or Netflix—including device-specific ones such as Apple Store (run by iOS devices) or Google Play (run by Android devices). The data were collected between May and June 2017 over the whole of France, and aggregated at the base station (BS) level. Because of their volume and scattered nature, some traffic from different applications were aggregated to common categories such as mail, gaming, news consumption (mainly newspapers outlets) or audio streaming (see electronic supplementary material, text and table S1). We merge the per-service traffic volume recorded in each BS coverage area with socio-economic indicators gathered from the 2014–2015 census, which include information about the income and population structure in each IRIS zone, i.e. the French sub-municipal statistical unit (see Methods). The combination of the two datasets is performed via an *areal interpolation* that maps mobile traffic over BS coverage areas into IRIS zones (figure 1).

Since our traffic data are collected by BS, they include app usage by residents of that area and users from other areas that visit that BS throughout the day. To link traffic data to the residents of a particular statistical area, we implemented a temporal consolidation of our data in which we only consider the mobile service usage recorded during the hours in which we can safely consider people to be at home, i.e. between 20.00 and 7.00 during weekdays (see Methods and electronic supplementary material, text).

The various mobile applications inherently entail very different traffic volumes: for instance, YouTube video streaming sessions consume much more data than Twitter messages. Therefore, plain traffic byte counts per inhabitant are not comparable across services and tend to conceal subtle differences in usage patterns, as exemplified in figure 1 and electronic supplementary material, S1. In order to bring patterns in the consumption of individual applications to the foreground, we use the revealed comparative advantage (RCA) [24] to normalize the aggregated traffic by IRIS area and service. RCA measures the ratio between the share of traffic generated by an application in a certain IRIS area and the same share computed in the whole country; it can thus reveal higher or lower relative adoptions of specific mobile services in a given area with respect to the national average.

**Figure 2.**
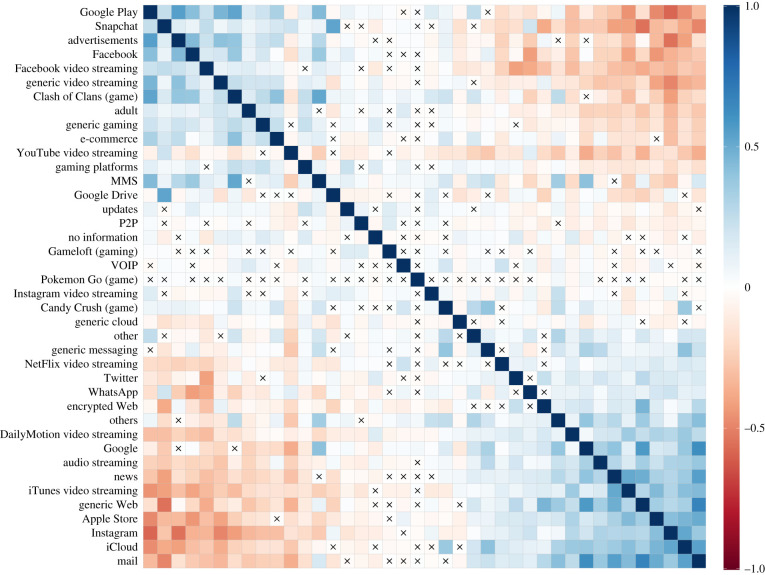
Correlation matrix of the consumption of each pair of mobile services, across all considered IRIS zone after RCA scaling. Variables are clustered using first principal component order. Non-significant coefficients are crossed out. MMS, Multimedia Messaging Service; P2P, peer to peer; VOIP, voice over Internet protocol.

The spatial, temporal and scale consolidation of the data outlined before allows a structure of correlations to be revealed in the usage of mobile service across geographical areas that was not recognized to date (figure 2). Specifically, previously observed strong correlations among different byte-count traffic flows [25] dissolve into a fabric of mild pairwise correlations and anti-correlations. We can clearly distinguish two groups of traffic flow RCAs which are loosely correlated within themselves and anticorrelated between them. We can easily recognize device-specific ones such as the Apple Store and iCloud on one of them and their counterpart (Google Play) in the other. Beyond that, the former seems to be dominated by more information apps (Google, news, mail), while the latter is composed of video-streaming traffic or gaming. Social media traffic is different also across both groups: while Instagram and Twitter traffic flow seems to be more correlated with the news and mail group, large Facebook or Snapchat usage co-occurs with generic video streaming and Google Play. Also gaming usage is different across groups, and is mainly concentrated in the group of high use of Facebook, Google Play and video streaming. The result highlights a pronounced spatial uniqueness in the consumption of each application, when relative usage is compared across different geographical units at a national scale.

In order to explore dependencies between such spatial diversity in mobile traffic and SES indicators, we gathered three demographic variables in each IRIS area from census data: (i) the median income, (ii) the ratio of people with a professional activity that requires higher education (or *higher education ratio*, for short, hereafter), and (iii) the Gini index of the income distribution, as a measure of local inequality. We model these three responses to try to explain them as a function of the relative traffic usage per category across areas. We use the population structure (i.e. population ratio by age ranges and immigrant ratio) as control variables in the framework of a generalized linear model weighted by the population counts in each area, with link functions specifically tailored to each response considered (Gamma regression with log link for median income; quasi-binomial regression with logit link and fractional response for higher education ratio; and Beta regression with logit link for local inequality). All the regressors are standardized prior to model fitting. As for the spatial autocorrelation, the distribution of the response variables as well as the dimension of the problem (11 000 observations of 40 covariates) make traditional approaches (spatial lag/error models and eigenvector selection for semi-parametric spatial filtering) computationally unfeasible. Thus, we developed a hybrid approach between a spatial error model and spatial filtering, implemented in two stages: in a first stage, the model is fitted without taking into account the spatial dimension, which produces spatially autocorrelated residual deviances; then, these are spatially lagged and re-introduced in a new fit as an additional auto-covariate. Our results show that this technique not only is much faster computationally but also successfully filters the spatial autocorrelation in the final model (as measured by the Moran-I value), producing stable estimates (see Methods for further details).

**Figure 3.**
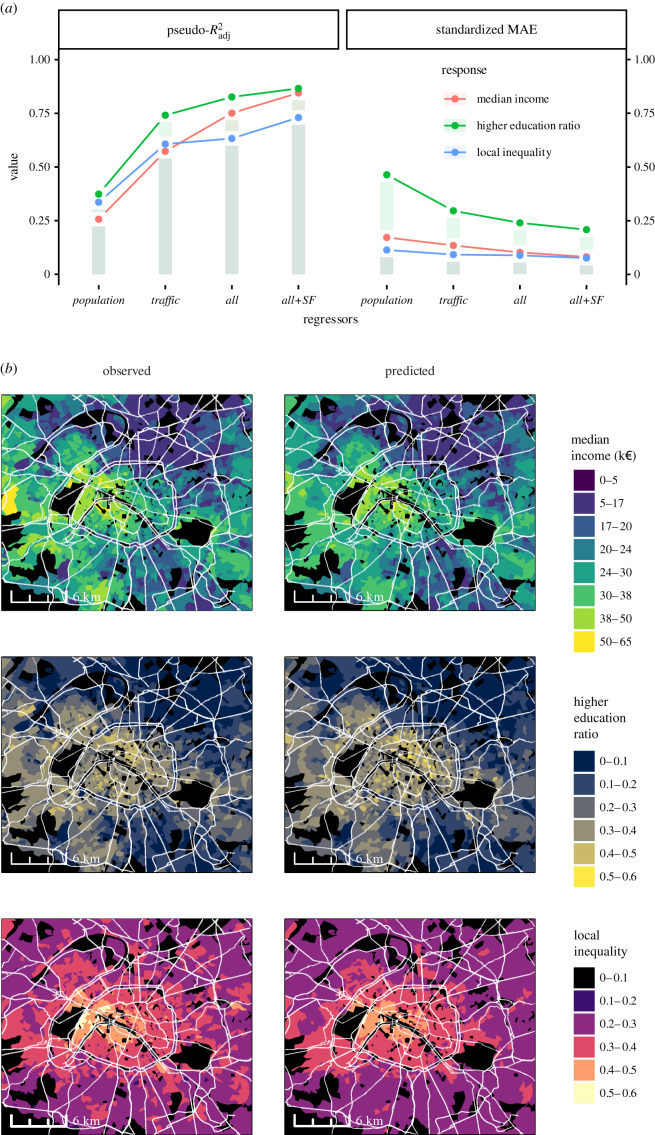
Regression results for the three SES responses considered, i.e. median income, higher education ratio and local inequality. (*a*) Performance metrics for each response and four different sets of regressors: (1) *population* structure variables, (2) *traffic* features, (3) both *population* and *traffic* features (*all*), and (4) *population* and *traffic* features combined with spatial filtering (*all+SF*). The left panel shows the adjusted pseudo-*R*^2^ obtained from the linear relationship between the observed versus predicted values. The right panel shows the standardized mean absolute error (MAE), computed as the MAE divided by the mean response. (*b*) Map of observed (left) versus predicted (right) responses using the best model (*all+SF*) in the Paris metropolitan area.

Figure 3*a* shows the quality of the regression on the three SES responses (i.e. median income, higher education ratio and local inequality) using four sets of predictors: population (control) variables, normalized mobile service traffic and both sets of variables without (*All*) and with (*All+SF*) spatial filtering. The left panel shows that the control variables alone explain a low ratio (in the 0.25–0.35 range) of the total variance, measured by an adjusted pseudo-*R*^2^, for the three SES models. On the other hand, mobile application traffic features alone significantly predict SES responses (with up to 0.74 of variance explained for the higher education ratio). Jointly considering population and traffic variables, as well as adding spatial filtering, further improves the result: ultimately, 0.73, 0.84 and 0.87 of the variance can be predicted for local inequality, median income and higher education ratio, respectively. The right panel in figure 3*a* shows the mean absolute error (MAE), standardized by the mean response, so that the three models can be compared. Notably, the best model in terms of explained variance is the worst in terms of relative MAE, and vice versa. This can be explained by the much larger variability that the higher education ratio presents in comparison with the other two SES responses. As a consequence, this model, despite being very reliable when it comes to capturing averages and general trends across spatial units (even for the traffic variables alone), is less suitable for point estimates than the others. The overall predictive power for these models is depicted in figure 3*b* for the Paris metropolitan area. The three SES responses are bucketed in fine-grained categories. Predicted values, on the right, show an excellent agreement with the observed ones.

**Figure 4.**
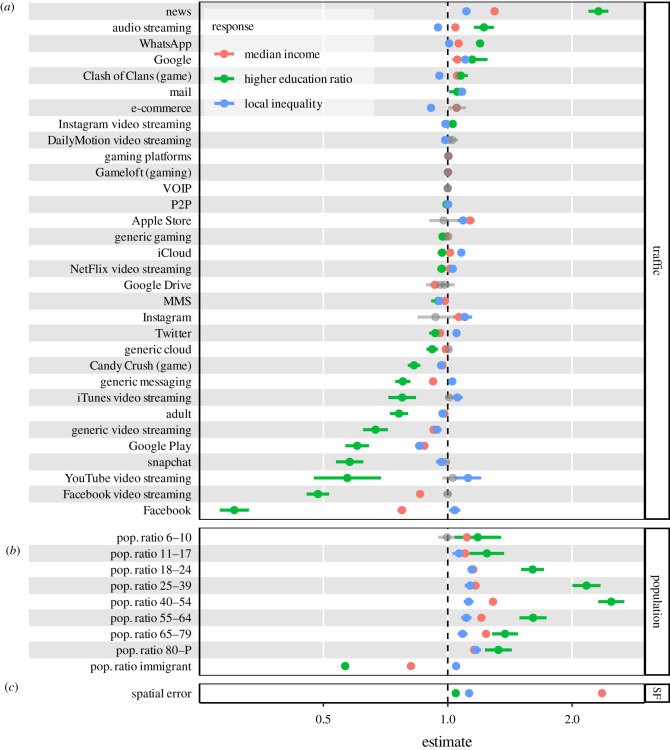
Relative effect sizes for the three SES responses considered: median income, higher education ratio and local inequality. Model estimates with 95% CIs are shown for traffic features (*a*), population variables (*b*) and the spatial term (*c*), in logarithmic scale. Non-significant estimates are greyed out. The model for the higher education ratio presents the stronger (positive and negative) effects overall. The median income response shows the higher spatial correlation. MMS, Multimedia Messaging Service; P2P, peer to peer; SF, spatial filtering; VOIP, voice over Internet protocol.

We compare the relative effect size of traffic variables, population control variables and spatial filtering (SF) for the best models (*all+SF*) in figure 4, with 95% confidence intervals (CIs). News and Facebook traffic stand out as key explanatory variables for all SES models, with especially high coefficients for the higher education ratio and median income. Their effect is however antithetical: a stronger usage of news applications positively correlates with income and education levels, whereas the increased usage of Facebook is associated with reduced income and education. Similar antagonistic behaviours are found in the specific groups of mobile services found in figure 2: for instance, a relatively higher consumption of WhatsApp, e-mail and audio streaming services is associated with higher income and education, but the increased use of Snapchat, video streaming or adult services has an opposite effect. Gaming also has mixed relationship with SES: while Candy Crush is more used in areas with low income and education ratio, the opposite happens for Clash of Clans. The large size effects identified for traffic variables suggest a deep usage gap of mobile phone data between different areas of income and educational attainment. On the other hand, traffic variables tend to have a different role when local inequality is concerned: as an example, audio and video streaming have reverse correlations (i.e. negative and positive, respectively) with this SES response. Especially good predictors of inequality are the adoption of iOS (i.e. Apple Store) devices in areas where income disparity is higher, and Android (i.e. Google Play) devices where the economic status of the population is instead more homogeneous. Putting together our results in figure 4, we can generally say that high-income and high-education areas have relatively more traffic on information-seeking tools (news, Google, mail), e-commerce and audio streaming, while areas with low SES indicators have higher relative traffic on social media activity and streaming (Facebook, YouTube, Snapchat).

Regarding population structure variables, most age groups show a similar positive effect on the responses, except for the higher education ratio, for which ages from 18 to 64 exhibit larger effects—as these are naturally the groups that had access to higher level education. On the other hand, the ratio of immigrants is associated with lower levels of median income and education, as well as higher levels of inequality, which can be expected. Remarkably, this pattern is consistent with the estimates for mobile services such as Facebook, generic messaging and Twitter. Finally, we quantify the relative importance of the spatial filtering in the same way as the rest of the variables in the model, although the method does not allow an actual spatial correlation to be estimated. In this sense, our estimates show that the spatial term is especially influential in the case of median income, but less important in the other responses.

## 3. Discussion

The data revolution has created an opportunity to scrutinize individual and collective behaviour at an unprecedented scale, detail and speed. We now have the opportunity to measure, monitor and predict relevant aspects of SES and growth in quasi-real time by using satellite images, social media or mobile phone data. More interestingly, some of these models relate socio-economic development to meaningful measures of human behaviour such as diversity, expressed opinions, purchases or the urban environment [7,8,10,13]. Thus, they can be used not only to monitor human development but also to understand the roots of SES and inequality. However, there seems to be a balance between predicting power and interpretability [13]. While machine learning models applied to satellite imagery and mobile phone data achieve typically high precision to explain SES [7], highly interpretable models based on diversity of mobility, purchases, content or other more interpretable metrics have less powerful explanatory power [8,10,13].

Our results show another dimension of human behaviour obtained from mobile phone data, i.e. digital usage can be used to achieve both high predicting power and interpretability of SES, even in developed countries. By just leveraging privacy-preserving aggregates of consumption of different services through mobile phones, we were able to have simple interpretable models for SES with high precision (approx. 80% of variance explained), larger than other models based on mobility diversity [8] or satellite imagery [13] for the same regions in France. Since our approach is complementary to these ones, there is a possibility that better precision can be obtained by combining our data with satellite imagery, for example. Finally, we found that the usage gap is partly drawn around the already observed iOS/Android operating system divide [26], with a positive correlation for iPhone users and a negative one for Android devices. More importantly, we took a step further, and revealed that the gap goes beyond plain platforms and roots deeply into the usage of different apps. We note that, to allow for demographic matching, we use the patterns of consumption for a specific time frame that are most likely to be produced by users when they are in their residential areas. The unobserved time window constitutes a limitation, in the sense that it would be possible for different demographic groups to present similar overall patterns of mobile consumption, but with a different distribution throughout the day. In such a case, this study would be detecting *when* services are consumed, instead of *which* services. However, the results from previous studies, as discussed above, as well as our robustness checks show that our results hold for different definitions of the observation period, such as weekends or earlier in the day.

The success of our models is based on a dramatic difference in mobile phone usage behaviours across groups of different SES during our observation window. The digital usage gap is so profound between low- and high-income or low- or high-education areas that it can be used to clearly distinguish between them or even identify the relative composition of these groups in a given area (Gini coefficient). High-income areas or those with higher education attainability show a more pronounced utilization of mobile devices to consume news, exchange e-mails, search for information or listen to music. At the same time, they display a reduced use of some social media platforms or video-streaming services. These results hold even when we control for age composition and other census variables such as an immigrant population. Although our models are equally accurate, the impact of the digital usage gap is more important for educational attainability. We can clearly see how regions that consume more Facebook content and less news have in general a lower fraction of the population with a higher education. This can be related to the two competing paradigms for online information consumption: the usage of traditional media versus social media platforms. Social media has reshaped news by facilitating the involvement of audiences, and thus boosting engagement and dissemination [27]. Platforms such as Facebook and YouTube have been identified as the major pathways to the increasing habit of using social media as a news source [28,29]. Even when perceived as unreliable, these platforms are used as ‘big outlets’ for convenience, especially by young adults [30]. However, since we control for age composition, this is not strictly an effect of generational differences of social media and news usage. Rather, it might be related to how less-educated people consume news: for instance, US adults who rely mostly on social media for news tend to have lower levels of education than those who mainly use several other platforms [31]. Another study in Chile found strong correlations between the socio-demographics of users and online news media content [21]. Given that polarization and spreading of misinformation is more likely on social media [19], our results could also be used to identify those populations and areas which could be more susceptible to these problems.

Following the Bourdieusian framework [32], we can assume that the practices of individuals in the field of mobile Internet highlight interrelations between economic resources and social positioning—and, probably, internalized abilities. For example, in the analysis of the digital activities of Italian youth according to their social background, Micheli [33] found that, while information seeking is positively correlated with the cultural capital of the students and the professional status of their parents, this is not the case for social media use. Adolescents from disadvantaged social backgrounds are more likely to actively participate in social media than adolescents from upper strata. Micheli's interpretive analysis of qualitative data indicates that upper-middle class students replicate their parents’ attitudes towards the Internet as a tool for personal enrichment.

Finally, it is worth noting that our results are based on a fully privacy-preserving analysis of mobile phone data. While other metrics based on user mobility and communications need individual or high-resolution data, our variables are constructed using aggregates of traffic at network BS. Such variables are fully compliant with the General Data Protection Regulation (GDPR), since they typically blend in a non-reversible way data generated by hundreds of users, hence they do not incorporate any personal information and hinder the possibility of de-anonymizing individual information. Also, they are compact enough to enable very large-scale analyses such as the one we carried out, and they are relatively simple to collect for mobile network operators, easing the permanent availability of statistics for longitudinal studies. More importantly, since our analysis is complementary to the ones using other dimensions of mobile phone data (mobility, diversity of communications), we believe our results will foster a new analysis in the future about the relationships between different aspects of access to information, human communication and mobility and their impact on human development and SES.

Although our results are descriptive and do not imply causal relations, we believe that our findings could be used to point to important and previously overlooked factors of socio-economic inequality whose causal effect may be further tested through carefully designed experiments, interventions or digital regulations. For example, the fact that low income or educational attainment is correlated with groups of services such as social media, video streaming or messaging could be used to devise successful holistic interventions to minimize their use and promote other mobile phone usages.

## 4. Material and methods

### 4.1. Mobile service traffic data

The network traffic dataset employed by our study comprises usage statistics of popular mobile applications. Data entries are recorded as the uplink (data transmitted by the user device) and downlink (data flowing to the user device) byte counts per service, at a temporal granularity of 5 min and aggregated by BS. The data were collected by Orange France within its own infrastructure during 1.5 months in May and June 2017. They describe the mobile behaviour of the whole Orange subscriber base in France, i.e. approximately 15 million individuals distributed over more than 550 000 km^2^ and served by over 25 000 BS. Usage statistics were collected by passive probes monitoring user sessions; the specific mobile service associated with each session was detected using deep packet inspection (DPI) and fingerprinting techniques tailored to specific traffic types (see electronic supplementary material, SI appendix for further details). The final dataset made available by the operator included the 40 services that generate the most traffic in the network, as detailed in electronic supplementary material, figure S1.

### 4.2. Geographical data and socio-economic indicators

We used geographical information and census data from the French Institut national de l’information géographique et forestière (IGN), which are publicly available in their web pages. For the geographical description, we downloaded the *Contours IRIS édition 2016* dataset, which defines a polygon in a Lambert-93 projection for each IRIS zone (i.e. aggregated unit for statistical information) in France, as well as an associated record containing the IRIS code, name and type among other information. For the population structure, we downloaded the *Population en 2015* dataset, which contains a description of the population structure by age group and other factors, such as socio-professional category and immigration. For the economic indicators, we downloaded the *Revenus, pauvreté et niveau de vie en 2014 (IRIS)* dataset, which contains a complete description of the income distribution deciles for residential IRIS zones. These are areas with more than 1000 inhabitants, and their population generally falls between 1800 and 5000. Indicators for areas with less than 1000 are not shared by the IGN for privacy reasons.

### 4.3. Areal consolidation

The coverage area of each BS in the Orange mobile network is modelled via a Voronoi tessellation that uses the BS location as the object positions on the geographical space. Such BS coverage areas have a different geometry from the IRIS zones for which income and population data are available; generally, coverage areas are much smaller than IRIS zones in urban centres, but the opposite occurs in the countryside and less populated regions of the country. To spatially consolidate the data, we adopted an *areal-weighted interpolation* procedure to transfer BS-level traffic counts into IRIS zones. As exemplified in figure 1, the principle is computing the intersection between the two spatial bases, and then creating a many-to-one mapping of BS coverage sub-areas to IRIS zones (i.e. determining which IRIS zones each BS coverage area intersects with) plus a set of associated areal weights (i.e. the surface fraction of original BS coverage area that falls into each BS sub-area). By assuming that mobile service traffic is evenly distributed within the BS coverage area, traffic counts for each BS sub-area are calculated as the areal weight multiplied by the total traffic recorded for the BS, for each service. Finally, the traffic counts for all relevant BS sub-areas are aggregated for each IRIS zone. After filtering out IRIS zones without economic indicators, we have mobile service traffic data for 11 806 IRIS zones (out of 49 404), which encompass all the main urban areas of France as depicted in electronic supplementary material, figure S2. Classified by their degree of urbanization (according to Eurostat), we find that 78% of the IRIS zones in the final dataset correspond to urban areas, 19% are peri-urban areas and 3% are rural areas.

### 4.4. Temporal consolidation

A mismatch between traffic and socio-economic datasets exists also in the temporal dimension, because of the inherent *mobile* nature of the consumption of applications on portable devices as opposed to the *static* character of census indicators. We resolve the discrepancy by only considering the mobile service usage that is most likely to be produced by users when they are at their locations of residence—which their socio-economic indicators also refer to. More precisely, we filter out weekends and French holidays (25 May and 5 June in the period considered), and we keep observations during home hours (from 20.00 to 7.00) on weekdays. There is evidence that app usage peaks from 20.00 [34], and that online consumption is more or less homogeneous throughout the day [35]. Although there could be important differences in traffic during the day for individuals, we believe that our aggregate consumption data by area are highly representative of the daily online consumption of the population of the area. As we show in the electronic supplementary material, SI appendix, our results are robust to the definition of home hours, and even hold for weekends, with no unobserved period.

### 4.5. Scale consolidation

Different mobile applications generate heterogeneous volumes of network traffic depending on the nature of the data transferred (e.g. video streaming creates a much higher load per session than messaging) and popularity (with widely adopted services producing a much higher demand than niche ones). This results in diverse scales for traffic counts across services, which can span several orders of magnitude, as observed in electronic supplementary material, figure S1. In addition, as shown in figure 1, raw byte counts are highly correlated across different mobile services, both spatially and temporally.

The scale mismatch and spatio-temporal correlation tend to hide differences in mobile service consumption. In order to give prominence to any such diversity, we aim at adopting a relative metric of the traffic with the property of being comparable across spatial zones and applications. Firstly, we consider the downlink byte counts for all services, which is aggregated on a hourly basis and normalized by census population. We then take the median values of the downlink bytes/inhabitant/hour during the whole 1.5-month observation period, for each IRIS zone and mobile service. Finally, we calculate the revealed comparative advantage (RCA) [24] as follows:

4.1 $${\mathrm{RCA}}_{ij}=\frac{T_{ij}/T_{i}}{T_{j}/T},$$

where *T*_*ij*_ is the median hourly traffic per inhabitant in zone *i* for application *j*; *T*_*i*_ is the median hourly traffic per inhabitant in zone *i* jointly generated by all considered applications; *T*_*j*_ is the median hourly traffic per inhabitant generated by service *j* in all zones at once; *T* is the median hourly traffic per inhabitant, aggregated over all zones and services. The index in equation (4.1) measures the proportion of traffic generated by a particular mobile application in a specific IRIS zone, normalized by the fraction of global (i.e. over all zones) traffic imputed to that same application. An *advantage* of service *j* is revealed in IRIS zone *i* if RCA_*ij*_ > 1, implying a higher-than-ordinary usage of service *i* in area *j*; conversely, if RCA_*ij*_ < 1, application *j* presents a *comparative disadvantage*, i.e. a reduced adoption with respect to the national average, in zone *i*. The metric allows all traffic features in a common unit to be measured, and reveals a structure of mild correlations and anti-correlations, shown in figure 2, which is instead concealed by uniform strong interdependence when considering raw byte counts.

### 4.6. Multicollinearity handling

The RCA transformation is a relative measure of importance, and, as such, we have to drop at least one variable to avoid a *perfect fit*, i.e. that every RCA_*ij*_ is an exact linear combination of remaining RCA_*ik*_, ∀ k≠i. We dropped a *no info* service, which gathers traffic generated by unknown applications.

To diagnose the presence of multicollinearity in the remaining set of variables, we compute the variance inflation factor (VIF) for each individual RCA_*ij*_ using the median income as the dependent variable. This method reports a high level of multicollinearity, with an average VIF of approximately 4 and a median VIF of approximately 27 across variables.

Therefore, we proceed to manually remove a few residual traffic categories and uninformative ones that are of little interest from the behavioural perspective. These are *Pokemon Go*, *other(s)*, *advertisements*, *updates*, *encrypted web* and *generic web*. After dropping these variables, 32 services remain, which are listed in figure 4; the same diagnostic run on the lasting variables reports average and median VIF of approximately 2, which corresponds to a low level of multicollinearity.

### 4.7. Regression models

We consider two socio-economic indicators in IRIS zones that are available in the public datasets, i.e. the median income and the ratio of people with a professional activity that requires higher education, or *higher education ratio* for short; in addition, we consider a third inequality indicator in the form of the Gini index computed from the income data (see the electronic supplementary material, SI appendix for further details). We model the dependency of the indicators on mobile service usage via a generalized linear model

4.2 $$g(E[y_{i}])=\alpha_{0}+\sum_{j}\beta_{j}\cdot {\mathrm{RCA}}_{ij}+\sum_{k}\gamma_{k}\cdot {\mathrm{POP}}_{k}+\delta \cdot {\mathrm{SP}}_{\mathrm{err}},$$

where $y_{i}\equiv \{\mathrm{income},\;\mathrm{education},\;\mathrm{inequality}\}$ for zone *i* is modelled after the RCA_*ij*_ values for each application *j*. POP_*k*_ are control variables from the population structure, i.e. the ratio of inhabitants in the 11–17, 18–24, 25–39, 40–54, 55–64, 65–79 and 80+ ranges, plus the ratio of the immigrant population. Both groups of regressors, RCA_*ij*_ and POP_*k*_, are standardized: scaled by the square root of the second raw sample moment of the whole group, so that the coefficient estimates and effect sizes across groups are comparable. The link function *g* is tailored to the distribution of each response.

— Median income is a positive-definite continuous response that can be modelled after a Gamma function. Thus, we perform Gamma regression with a log link, and estimates are interpreted as a means ratio.
— Higher education ratio is the proportion of people with a professional activity that requires higher education, which is a counting process that may be overdispersed. Thus, we define a fractional model, i.e. a quasi-binomial regression with a logit link and fractional response, and estimates are interpreted as an odds ratio.
— Local inequality is measured with the Gini coefficient, which can be modelled after a Beta distribution. Thus, we perform a Beta regression with a logit link, and estimates are interpreted as an odds ratio.

Finally, the high Moran-I value for each response (0.72, 0.81 and 0.74, respectively; see electronic supplementary material, table S2) justifies the use of a spatial model. Therefore, SP_err_ is a variable created to filter the spatial correlation, and is defined as the spatially lagged residual deviance of the rest of the model

4.3 $${\mathrm{SP}}_{\mathrm{err}}=Wr_{i},$$

where *W* is the row-standardized matrix of queen-contiguity spatial weights and *r*_*i*_ is the deviance residuals for an initial fit with the rest of the variables involved (see the electronic supplementary material, appendix for further details).

These models are thus fitted in four stages: (i) a reference fit with the population variables alone, which serves as a null model; (ii) a second fit with traffic variables alone, to explore their explanatory power; (iii) a complete model with both traffic and population variables; and (iv) a final model that performs spatial filtering by taking the deviance residuals *r*_*i*_ from (iii), which show spatial correlation, and incorporating them as SP_err_ in a new fit. We checked that point estimates for RCA_*ij*_ and POP_*k*_ in (iii) and (iv) are very similar, but (iv) succeeds in filtering out the spatial correlation (*p* < 0.001 for the Moran-I test), thus producing better results and more precise and stable coefficients (see electronic supplementary material, figures S5–S7 and tables S3–S6).

## Data accessibility

The data that support the findings of this study are available from Orange, but restrictions apply to the availability of these data, which were used under licence for the current study, and so are not publicly available. Data are however available from the authors upon reasonable request and with permission of Orange.
